# Molecular Imaging to Identify Tumor Recurrence Following Chemoradiation in a Hostile Surgical Environment

**DOI:** 10.2310/7290.2014.00051

**Published:** 2014

**Authors:** Olugbenga T. Okusanya, Charuhas Deshpande, Eduardo M. Barbosa, Charu Aggarwal, Charles B. Simone, Jack Jiang, Ryan Judy, Elizabeth DeJesus, Steve Albelda, Shuming Nie, Philip S. Low, Sunil Singhal

**Affiliations:** Division of Thoracic Surgery, Department of Surgery, Department of Pathology, Department of Radiology, Division of Hematology Oncology, Department of Medicine and Department of Radiation Oncology, University of Pennsylvania, Perelman School of Medicine, Philadelphia, PA; Departments of Biomedical Engineering and Chemistry, Emory University, Atlanta, GA; and Department of Chemistry, Purdue University, West Lafayette, IN

## Abstract

Surgical biopsy of potential tumor recurrence is a common challenge facing oncologists, surgeons, and cancer patients. Imaging modalities have limited ability to accurately detect recurrent cancer in fields affected by previous surgery, chemotherapy, or radiation. However, definitive tissue diagnosis is often needed to initiate treatment and to direct therapy. We sought to determine if a targeted fluorescent intraoperative molecular imaging technique could be applied in a clinical setting to assist a surgical biopsy in a “hostile” field. We describe the use of a folate-fluorescein conjugate to direct the biopsy of a suspected recurrent lung adenocarcinoma invading the mediastinum that had been previously treated with chemoradiation. We found that intraoperative imaging allowed the identification of small viable tumor deposits that were otherwise indistinguishable from scar and necrosis. Our operative observations were confirmed by histology, fluorescence microscopy, and immunohistochemistry. Our results demonstrate one possible application and clinical value of intraoperative molecular imaging.

*INNOVATIONS* in drug development and radiotherapy allow cancer patients to survive longer in disease remission.^1–5^ In these patients, monitoring for recurrence in fields affected by fibrosis, necrosis, and inflammation is a major clinical challenge as anatomic and metabolic imaging modalities have limited value in these situations.^6,7^ Thus, patients with suspected recurrences are often referred for definitive surgical biopsy before reinitiating therapy. These operations are challenging, with poor diagnostic yield due to the “hostile field” formed by scar and tissue necrosis. Others have shown that targeted molecular imaging probes can be used intraoperatively to detect cancer.^8,9^ Little is known about using this approach in a complex clinical situation and whether molecular imaging can be used to assist surgical biopsies in hostile fields. Here we describe an intraoperative molecular imaging strategy that uses a fluorescent tumor-specific ligand to perform a surgical biopsy in a hostile anterior mediastinum created by previous chemoradiation therapy.

## Methods

This study was performed after informed consent and approval by the Institutional Review Board (IRB) at the University of Pennsylvania were obtained. The patient was enrolled in the trial via written consent, which was also approved by the IRB. This report is a subset of a larger clinical trial registered at ClinicalTrials.gov under identifier NCT01778920.

To obtain tissue contrast, the patient was injected intravenously with a clinical grade folate receptor (FOLR)1 targeted optical fluorescent contrast agent (0.1 mg/kg folate-fluorescein) 4 hours prior to surgery (courtesy of On Target Laboratories Inc., West Lafayette, IN). Our intraoperative system was composed of a light source (excitation 490 nm) and two cameras (BioVision Inc., Exton, PA, and BioMediCon Inc., Moorestown, NJ).^10^ The cameras shared one lens, and the signal was split with a dichroic mirror. One camera captured all conventional white light emission, and the other camera had a filter behind the lens to capture only 520 nm light emission. To quantitate the tissue fluorescence, we used region of interest software and the HeatMap plugin within *ImageJ* (public domain free software developed by the National Institutes of Health, Bethesda, MD). Background readings were taken from intercostal muscle to generate a signal to noise ratio. All readings were done in quadruplicate. Fluorescence microscopy was performed using an Olympus IX51 fluorescent microscope equipped with a fluorescein-specific filter set (Chroma 49012). FOLR1 immunohistochemical staining was performed using the monoclonal mouse antihuman antibody Mab343 (Morphotek Inc., Exton, PA).

## Results

**Figure 1.**
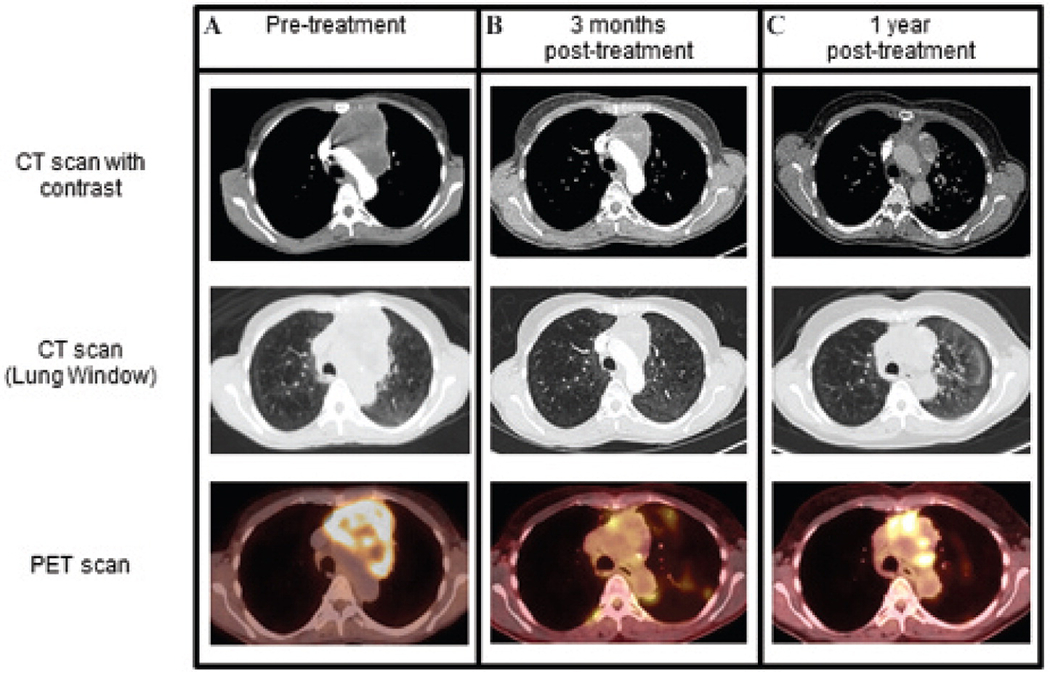
Imaging presentation of an anterior mediastinal mass in patient D.H. *A*, On initial imaging, the patient presented with an anterior mediastinal mass with heterogeneous enhancement that measured 8.6 × 7.8 cm in size (upper CT image) (maximum SUV 10.7, lower [^18^F]FDG PET image). The great vessels were displaced medially from the left side, and the upper lobe branch of the left pulmonary artery was displaced inferolaterally. *B*, After chemoradiation, surveillance imaging demonstrated a reduction in the mass to 5.6 × 3.1 cm (upper CT image), with only a small rim of increased tracer uptake along the right medial and inferior margins and a maximal SUV of 6.1 (lower fusion [^18^F]FDG PET/CT image). Given the favorable response, further therapy was deferred. *C*, One year later, there was an increase in the [^18^F]FDG uptake in the medial and inferior aspects of the anterior mediastinal mass (SUV maximum 8.7, lower fusion [^18^F]FDG PET/CT image), suspicious for tumor recurrence.

D.H. was a 54-year-old patient who presented with 3 months of a persistent irritation to the posterior pharynx. The patient was an office worker, a 15-pack-year smoker, and otherwise healthy; his Eastern Cooperative Oncology Group (ECOG) performance status was 0, with no family history of cancer. An initial chest x-ray showed a large left superior mediastinal mass extending from the aortic arch to the left pulmonary hilum. Laboratory studies showed lactate dehydrogenase of 221 U/L (normal 98–192 U/L), with all other tumor markers (α-fetoprotein, β-human chorionic gonadotropin, antiacetylcholinesterase antibody) within normal limits. An intravenous contrast-enhanced computed tomographic (CT) scan demonstrated an 8.6 cm anterior mediastinal mass with heterogeneous enhancement and medial displacement of the great vessels (Figure 1A).

**Figure 2.**
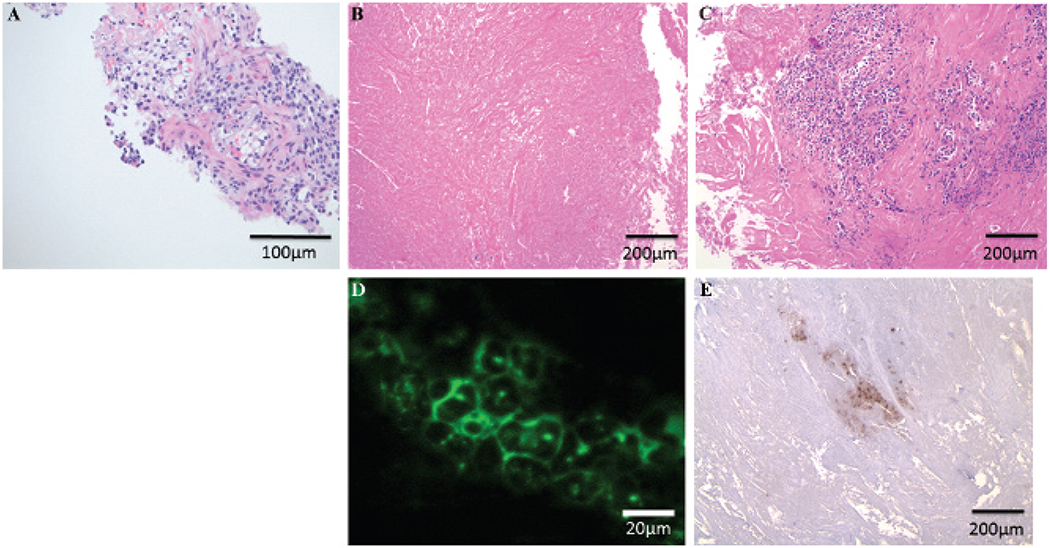
Pathologic examination of tissues obtained from patient D.H. *A*, Initial transthoracic needle aspiration obtained a limited tissue sample. The overall morphology and immunostaining (hematoxilin and eosin [H&E], ×20 original magnification) favored a malignant epithelioid neoplasm with clear cell features and suggested a poorly differentiated adenocarcinoma. *B*, One year later, during the surgical biopsy, multiple samples revealed extensive necrosis and fibrous tissue and no evidence of malignant cells (H&E, ×10 original magnification). *C*, By using molecular imaging for FOLR1, three precise biopsies discovered small foci of tumor cells (< 0.4 cm) surrounded by fibrous tissue (H&E, ×10 original magnification). *D*, Epifluorescence microscopy demonstrated that these tumor cells were fluorescent when excited with a 490 nm laser (×63 original magnification). *E*, Immunostaining confirmed that these malignant cells expressed FOLR1 (H&E, ×10 original magnification).

Transthoracic needle aspiration revealed malignant cells. Immunohistochemistry showed that tumor cells expressed cytokeratin 7, pancytokeratin, and MOC-31. They were focally weakly positive for CK20, thyroid transcription factor (TTF-1), c-Kit, and spalt-like transcription factor 4 (SALL4). They did not express CD30, α-fetoprotein, p63, placental alkaline phosphatase (PLAP), surfactant B, napsin-A, CD10, pax-8, thyroglobulin, or renal cell carcinoma. Histologic analysis identified a poorly differentiated cancer with clear cell features and nuclear atypia most consistent with an adenocarcinoma of primary lung origin (Figure 2A). Molecular analysis was negative for epidermal growth factor receptor and *KRAS* mutation. There was no *ALK* rearrangement on fluorescence in situ hybridization.

A positron emission tomographic (PET) scan showed the mass to have a maximum standardized uptake value (SUV) of 10.7 with no [^18^F]fluorodeoxyglucose ([^18^F]FDG) uptake of the mediastinal lymph nodes and no evidence of distant disease (see Figure 1A). Magnetic resonance imaging (MRI) of the brain was unremarkable. Clinically, the patient was designated to have stage IIIA (T4N0M0) pulmonary adenocarcinoma. Due to mediastinal invasion, the patient was deemed a poor surgical candidate.

D.H. was treated with 37 fractions using intensity-modulated radiotherapy delivering 180 cGy daily over 7½ weeks to a total dose of 6,660 cGy to the anterior mediastinal mass and left hilum with a seven-field technique. The patient concurrently received 50 mg/m^2^ of cisplatin and 50 mg/m^2^ of etoposide in four cycles over 36 days. D.H. tolerated the therapies well and had minor side effects of a left eyelid droop, anemia, grade 1 esophagitis, and grade 2 dermatitis, which all resolved with conservative management.

One month after completing therapy, D.H. underwent computed tomography (CT), which showed a partial response by Response Evaluation Criteria in Solid Tumors (RECIST) criteria. The tumor had reduced from a maximum tumor dimension of 8.6 cm to 5.0 cm (Figure 1B). A PET scan showed that the maximum SUV had decreased from 10.7 to 6.1 (see Figure 1B). A multi-disciplinary discussion regarding the favorable early response led to a decision to monitor for recurrence and defer further treatment. The patient was scheduled for surveillance imaging every 3 months.

One year later, routine PET/CT showed an increase in the size of the mediastinal mass from 5.0 cm to 5.7 cm, and the maximal SUV had increased from 6.1 to 8.7 (Figure 1C). Due to the high suspicion of recurrence, tissue diagnosis was sought to characterize the mass, perform molecular analyses, and aid in the selection of appropriate therapy. Attempts at a transthoracic needle aspiration were not diagnostic. Thoracic surgery was consulted for an incisional biopsy.

The surgical team was concerned about the significant radiation changes around the mediastinum and paramediastinal lung parenchyma. The patient was offered a left anterior mediastinotomy; however, the patient was informed that the diagnostic yield could be limited due to mediastinal scarring, insufficient tissue, and sampling error. The patient was approved for a pilot study of intraoperative molecular optical imaging. Since the initial tumor was a known pulmonary adenocarcinoma, which have upregulated levels of FOLR1, the patient was imaged using a folate fluorescein conjugated molecular imaging contrast agent.

**Figure 3.**
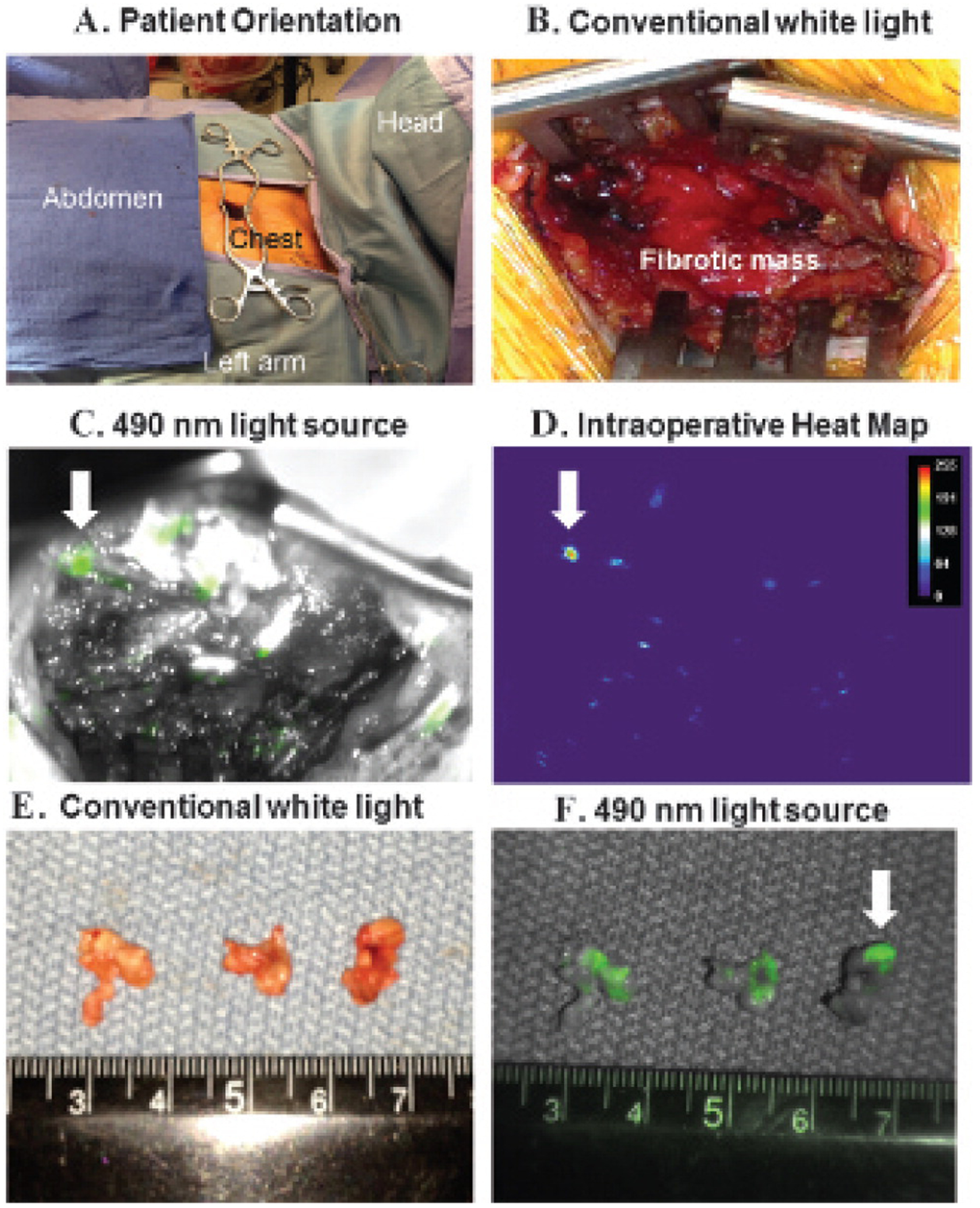
Intraoperative images from the surgical biopsy of the anterior mediastinal mass. *A*, The patient was oriented with the head to the right and a 2 cm incision over the left chest just lateral to the left border of the sternum in the second intercostal space. *B*, After dissecting the pectoralis and intercostal muscles, the surgeon encountered a dense fibrotic mass that required a scalpel to penetrate. Multiple surgical biopsies did not reveal malignant cells. *C*, The incision was illuminated with a 490 nm light source and revealed several small foci of fluorescent deposits in the wound. The largest suspicious tumor deposit is marked with a *white arrow*. *D*, Heat map analysis confirmed several areas of the mediastinal mass that fluoresced from the targeted FOLR1 contrast agent. *E*, Three-subcentimeter tissue specimens were taken with a biopsy forceps. On gross inspection, the three specimens appeared homogeneous and not different from the previous biopsies. *F*, However, the specimens were reimaged ex vivo and fluoresced; thus, this gave the surgical team a high degree of confidence that it had located tumor cells in the chest.

At the time of surgery, the patient was injected with 0.1 mg/kg of a FOLR1-targeted optical contrast agent. After induction, the mediastinum was accessed through a 2 cm incision in the second intercostal space lateral to the left sternal border (Figure 3A). After dissecting the pectoralis muscle and intercostal muscle, a solid, dense, fibrous tissue was encountered, and there was no delineation between tumor and nonviable tissue (Figure 3B). To obtain a biopsy, a sharp No.15 scalpel was needed to excise the solid mass. The operative field became bloody due to the neovascular ingrowth secondary to previous radiation therapy. Four biopsies (total 200 g) were sequentially obtained over 40 minutes. A lung pathologist was consulted intraoperatively for frozen-section analysis. Twelve separate sections were prepared, and each was individually reviewed. Rapid review revealed fibrosis with no tumor cells (Figure 2B). The surgical team felt that it had obtained a representative sampling, and the PET imaging likely represented inflammation.

At this time, a fluorescence imaging device was sterilely draped to image the surgical wound. Molecular imaging required less than 1 minute and identified several microscopic foci of bright fluorescence encased by the dense fibrosis (Figure 3C). Although several areas of glare were noted, only areas that fluoresced green on imaging were considered positive. A heat map confirmed increased fluorescence from these minute deposits (Figure 3D). A signal to noise ratio demonstrated 2.8-fold more fluorescence from the suspicious foci compared to the adjacent tissue. Three-subcentimeter excisional biopsies based on size, intensity, and location in the surgical field were performed guided by fluorescence. On gross examination ex vivo, these specimens were homogeneous and did not appear different from the previous biopsies (Figure 3E). However, molecular imaging confirmed fluorescence from portions of each biopsy (Figure 3F). Frozen sections identified small nests (< 0.4 cm) of tumor cells surrounded by dense scar tissue (Figure 2C). Microscopy showed clusters of fluorescent tumor cells when excited with a 490 nm laser (Figure 2D). The incision was closed, and the operation was concluded without injury to mediastinal structures, extension of the incision, or the need to remove a costal cartilage. The patient was discharged home.

Final pathologic review confirmed the malignant cells to be consistent with the patient’s diagnosis of a poorly differentiated lung adenocarcinoma. Staining was positive for pancytokeratin, CK7, and MOC-31 but focally weakly positive for CK20, TTF-1, c-Kit, and SALL4. Further staining of the tumor slides confirmed FOLR1 expression on the tumor cells (Figure 2E).

## Discussion

Over the last decade, advances in chemotherapy, immunotherapy, and radiation therapy have allowed oncologists to gain superior remission of solid tumors.^1–5^ However, a major challenge is an accurate approach to monitor and diagnose recurrent disease. For example, in this case, the 1-year interval scan demonstrated markedly increased [^18^F]FDG uptake in the anterior mediastinum. However, despite multiple biopsies, over 90% of the tumor was fibrotic and inflammatory; thus, the [^18^F]FDG grossly overestimated the degree of tumor recurrence.

Given the new targeted therapeutic options and the importance of personalized treatments, it is increasingly important to determine if tumor recurrence has occurred and to obtain tissue for molecular analyses. Radiologists can use CT to identify suspicious mass-like structures; however, this lacks anatomic detail and functional information. [^18^F]FDG-PET lacks the resolution to perform image-guided biopsies. Furthermore, PET can overestimate disease, as demonstrated by this case. Surgeons are reluctant to operate and perform biopsies in areas of previous radiation therapy or chemotherapy because they eliminate natural tissue borders and replace them with dense scar, adhesions, and thick fibrosis. Overzealous dissection can lead to inadvertent injury to normal structures. Surgical biopsy has no guarantee of uncovering a small focus of residual disease and has a sensitivity ranging from 30 to 70% depending on the amount of tissue reaction and damage.^11–13^ Surgeons use intraoperative consultation of pathologists; however, frozen-section analysis presents its own set of difficulties, including technical challenges of freezing tissues, artifacts, cost, lack of real-time data, loss of tissue in smaller specimens for permanent section diagnosis, and human and sampling error. Thus, adjuncts to intraoperative tissue biopsy in these so-called “hostile surgical fields” would be valuable.

We employed molecular optical imaging to identify and biopsy a microscopic proliferation of tumor cells within a large fibrotic mass. Our targeted contrast dye was linked to folate. Folate (molecular weight 440 kDa) plays a key role in metabolic processes involved in DNA and ribonucleic acid (RNA) synthesis, epigenetic processes, cellular proliferation, and survival.^14^ There are four members of the folate receptor (FOLR1–4) family, although only FOLR1 and FOLR2 bind folate with high affinity.^15–17^ FOLR1 is expressed at the luminal surface of polarized epithelial cells; thus, it does not typically bind serum folate. However, FOLR1 is also expressed at high levels (1–3 million receptors/cancer cell) on approximately 74 to 85% of lung adenocarcinomas.^18–20^ Increased FOLR1 expression on neoplastic cells likely arises as a means to compete for low serum folate concentrations (≈ 2 × 10^−8^ M) as folate is essential to support the increased DNA synthesis machinery in rapidly dividing cancer cells. In the future, as whole body radiolabeled folate scans become available, the intraoperative value of folate imaging could be determined preoperatively.^21^ Thus, FOLR1 provides a reasonable molecular target on pulmonary adenocarcinomas for diagnostic purposes.

Intraoperative optical imaging provides unique advantages that are not available with other intraoperative modalities. First, it does not require ionizing radiation. Thus, this technology is safe for patients and the personnel performing the procedure. Second, although optical probes have limited penetration depths due to tissue scattering and blood absorption, the lesions are surgically exposed and can be easily visualized. Alternative particles that permit deeper tissue visualization require higher excitation energy and risk desiccating tissues. Lastly, optical images are easy to interpret in real time during surgery.

We acknowledge that major limitations to fluorescein in human tissues are tissue scattering, absorption properties, and autofluorescence in the visible spectrum. Future targeted tracers with longer wavelengths (i.e., near-infrared 700–900 nm) will overcome these shortcomings but are not approved by the Food and Drug Administration or clinically available at this time. For superficial tumors at the organ surface, however, fluorescein does retain clinical value.

The implications of intraoperative molecular imaging are broad.^22^ The value of this technology is to draw attention to tissues that would otherwise be missed by simple visual or tactile inspection. This improves intraoperative staging and removal of residual disease.^23^ Cytoreductive surgery may become more thorough for many cancers, such as ovarian carcinoma, sarcomas, and malignant mesothelioma. For minimally invasive and robotic operations, including colonoscopy and esophagoscopy, where the surgeon has no benefit of manual palpation, tumor fluorescence may improve identification of tumor deposits. Finally, as the full capabilities of this approach are understood, the “real-time” aspects of tumor fluorescence imaging may provide surgeons with a tool to improve decision making in the operating room in a very rapid and cost-efficient manner.
